# ECOLOGICAL MOMENTARY ASSESSMENT AND MACHINE LEARNING FOR PREDICTING DEPRESSION AMONG OLDER ADULTS

**DOI:** 10.1093/geroni/igae098.2673

**Published:** 2024-12-31

**Authors:** Nan Jiang, Ke Chen, Yexuan Xiao, Vivian Weiqun Lou

**Affiliations:** Tsinghua University, Beijing, Beijing, China (People’s Republic); Tsinghua University, Beijing, Beijing, China (People’s Republic); Tsinghua University, Beijing, Beijing, China (People’s Republic); The University of Hong Kong, Hong Kong, China (People’s Republic)

## Abstract

Depression represents a significant global health concern, particularly among older populations. While numerous studies have focused on investigating chronic stressors, the precise predictive impact of daily experiences on depression remains uncertain. The objective of this study was to assess the extent to which mood fluctuations, daily stressors, contextual stressful events, and psychophysiological responses experienced by older adults with functional decline may predict later short- and long-term depression. This study employed a combination of longitudinal investigation and ecological momentary assessment (EMA) to collect data on the mood status and stressful events of older adults with functional limitation. Data were gathered 2 times per day over a 14-day period and followed up with a revisit one year later. To predict long-term depression, the study explored several approaches using machine learning techniques.These included analyzing chronic stress baseline data (baseline approach), examining dynamic patterns of mood states and stressful events through EMA approach, and combining baseline data with dynamic patterns (EMA plus baseline approach). A total of 166 older adults participated in the study. The baseline plus EMA approaches showed better performance than the baseline and the EMA approaches at 1-year follow-up (area under the receiver operating characteristic curve [AUC], 0.798; 95%CI: 0.599，0.932) on the prediction of depression. The results underscore the significance of contextual risk factors encountered by older adults during different stages. Leveraging machine learning techniques could enhance the identification of older adults at risk and contribute to the creation of personalized, process-based early prevention programs aimed at mitigating future depression.

